# Supplementary material for: Anomalous thickness dependence of Curie temperature in air-stable two-dimensional ferromagnetic 1T-CrTe2 grown by chemical vapor deposition
Source: Nat Commun. 2021 Feb 5;12:809. doi: 10.1038/s41467-021-21072-z (PMC7864961; doi:10.1038/s41467-021-21072-z)
Supplement: Supplementary file 1 — Supplementary Information [file 41467_2021_21072_MOESM1_ESM.pdf]

Supplementary Information

Anomalous thickness-dependence of Curie temperature in air-stable two-dimensional ferromagnetic 1T-$CrTe_2$ grown by chemical vapor deposition

Meng *et al.*

Supplementary Figures

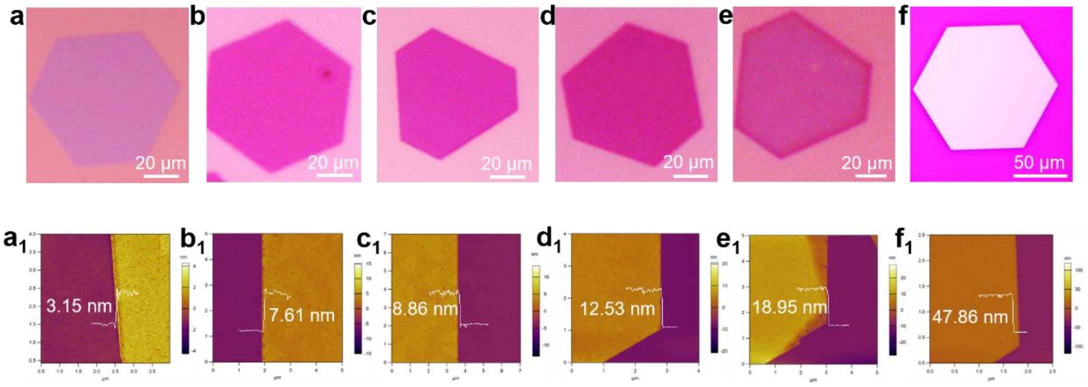

Supplementary Figure 1. Optical microscopy and corresponding AFM images of CVD grown 1T-$CrTe_2$ at different temperatures with various thickness. (a, b, c, d, e, f) OM images shown the 1T-$CrTe_2$ crystals with the thickness of 3.2 nm, 7.6 nm, 8.9 nm, 12.5 nm, 19.0 nm and 47.9 nm (a_1, b_1, c_1, d_1, e_1, f_1), respectively. The growth temperature was set at 976 K, 983 K, 985 K, 988 K, 991 K and 993 K, respectively.

We selected $CrCl_2$ as the Cr source due to its lower melting point compared to that of $CrCl_3$ and Cr_2O_3, that can lower the growth temperature. The uniform optical contrast from OM images indicates high uniformity of the synthesized crystals (Supplementary Figure 1). The thickness of the resulting sample is very sensitive to the growth temperature. Similar observations were previously reported in the growth of $NiTe_2$[1].

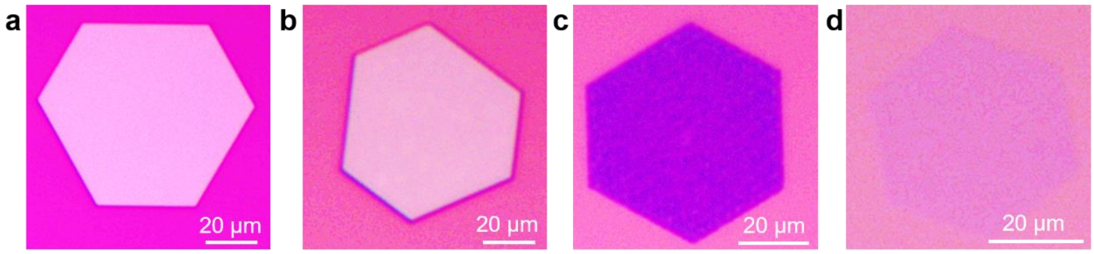

Supplementary Figure 2. OM images of CVD grown 1T-$CrTe_2$ under different atmospheric conditions. (a) The growth temperature was set at 983 K with 200 sccm pure argon as reaction atmosphere. (b - d) Grown under the same reaction condition as "a" but with additional hydrogen of 1 sccm, 5 sccm and 10 sccm, respectively. H_2 is used to reduce sample thickness via slow-etching. As shown, the optical contrast is reduced when the hydrogen concentration increases. When the hydrogen concentration is higher than 10 %, no sample was synthesized.

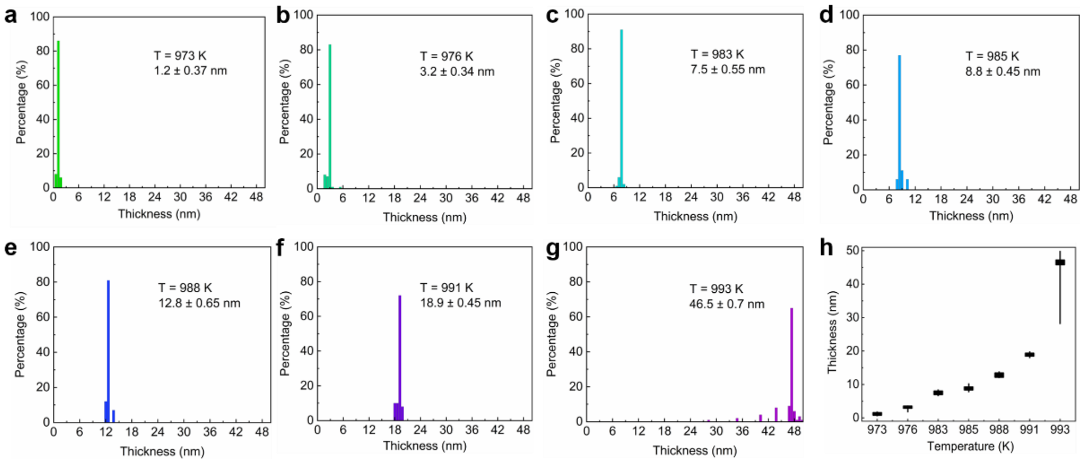

Supplementary Figure 3. The thickness histogram distributions of 1T-$CrTe_2$ crystals. (a - g) The samples were synthesized at the temperature of 973, 976, 983, 985, 988, 991 and 993 K, respectively. (h) The sample thickness as a function of growth temperature. The black line and the black rectangle indicate the range of the thickness and the averaged thickness of the samples grown at a given temperature, respectively. The result of each statistical diagram is calculated from five batches of growth at the same temperature.

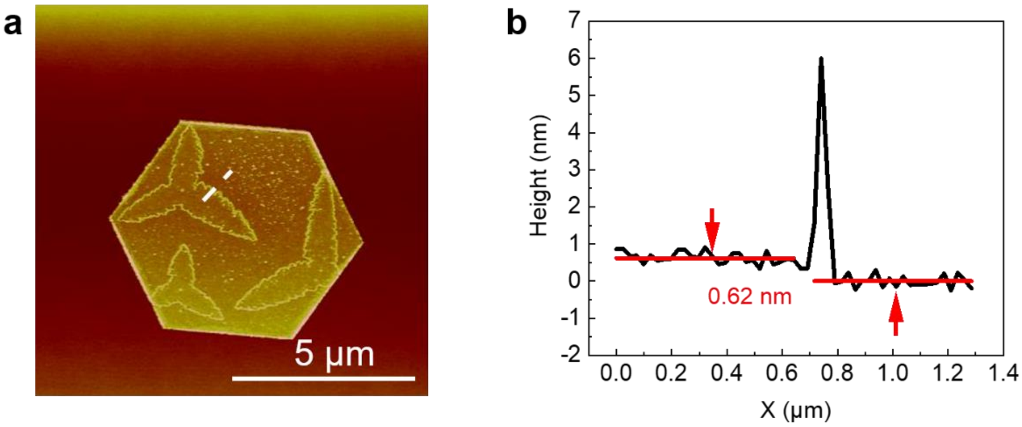

Supplementary Figure 4. AFM image and height profile of stepped 1T-$CrTe_2$ flakes. (a) AFM height topography of a stacked 1T-$CrTe_2$ single crystal. (b) Height profile along the white dashed line in (a), shown a thickness of 0.6 nm.

The thickness data from AFM can not only be mutually verified with that from cross-sectional STEM-HAADF, but also be used to confirm the layered nature of 1T-$CrTe_2$. In order to give a clear evidence, we found some samples with the top layer not completely covered, which has also been observed in the growth of other 2D materials[2,3]. The origin of multiple pits with trigonal symmetry may probably due to the layer by layer growth mechanism. The height profile extracted from AFM is about 0.62 nm which is comparable to that from cross-sectional STEM-HAADF and is in good accordance with the thickness of the unit cell of the 1T-$CrTe_2$ crystal[4].

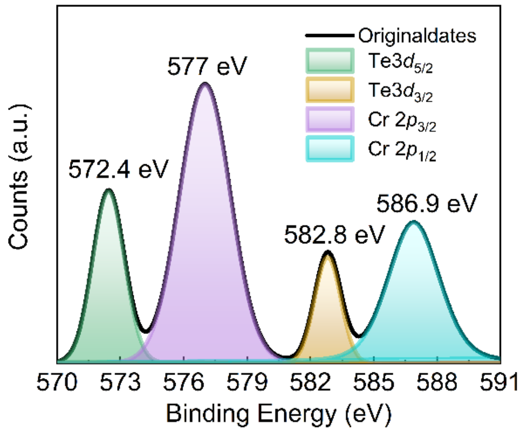

Supplementary Figure 5. XPS characterization of as-grown 1T-$CrTe_2$.

According to the XPS measurements, the binding energies for the Te 3$d_{5/2}$ and 3$d_{3/2}$ doublets are 574.4 eV and 582.8 eV, and those for the Cr 3$p_{3/2}$ and 3$p_{1/2}$ are 577.0 and 586.9 eV, respectively. The elemental analysis gives the empirical formula of $CrTe_2$.

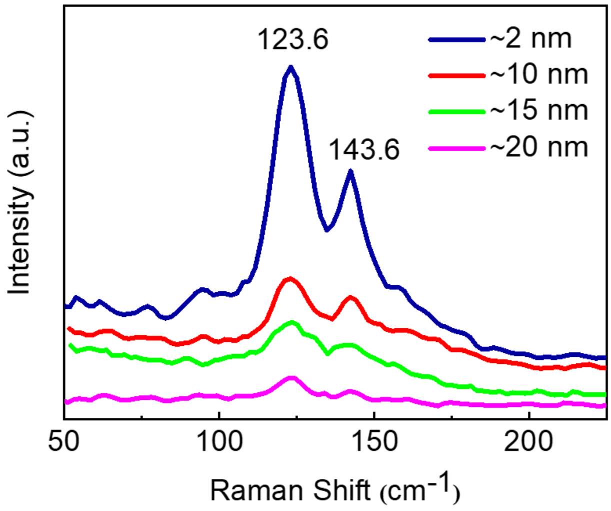

Supplementary Figure 6. Thickness-dependent Raman spectrum of 1T-$CrTe_2$ flakes in ambient atmosphere.

The Raman spectrum shows that there are two main characteristic peaks for 1T-$CrTe_2$, each at 123.6 cm^{-1} (E_{2g}) and 143.6 cm^{-1} (A_{1g})[5]. Thickness-dependent Raman measurement suggests that both modes show no obvious shift with increasing thickness, which is similar to that of VTe_2[6]. Additionally, the Raman intensity is monotonically decreasing with increasing thickness from 2.0 nm to 20.0 nm, providing an intuitive method for qualitative evaluation of sample thickness.

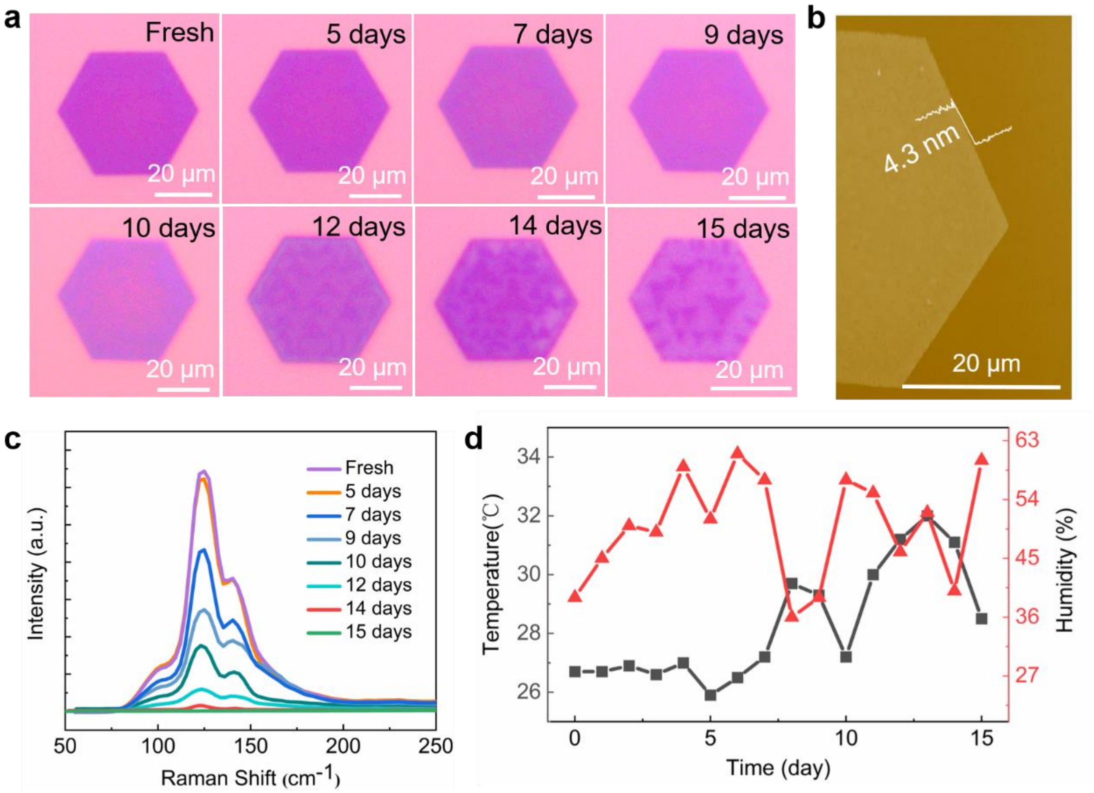

Supplementary Figure 7. Environmental stability investigations. (a) Optical images of 1T-$CrTe_2$ samples exposed in the atmosphere for 0 day, 5 days, 7 days, 9 days, 10 days, 12 days, 14 days and 15 days, respectively. (b) AFM image of the sample being tested. (c) The corresponding Raman spectrum from a. (d) Humidity and temperature data during the test.

After 5 days exposure in air, the sample has unchanged color, morphology and the Raman intensity are comparable to that of the fresh one. When the exposure time extends to more than 10 days, the color and morphology of 1T-$CrTe_2$ become lighter and rugged. The corresponding Raman signals decrease in intensity and ultimately vanish. Finally, after 15 days air-exposure, no Raman signals are detected, which indicated that the sample has degenerated. Ambient temperature and humidity conditions may have a significant impact on the stability investigations, thus we have also recorded the data simultaneously.

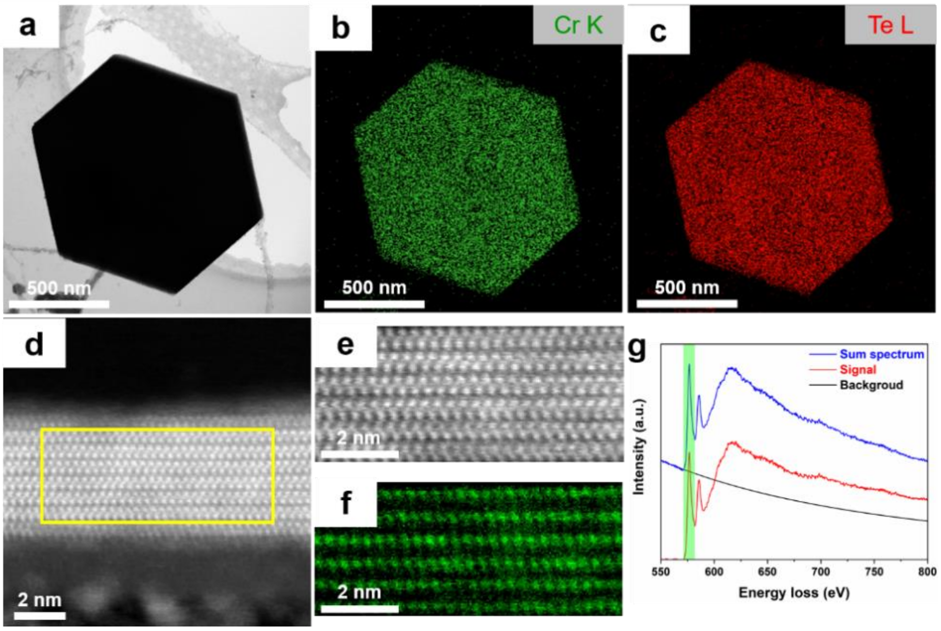

Supplementary Figure 8. The elemental analysis of the hexagonal 1T-$CrTe_2$ Sample. (a) The low magnification TEM image. (b, c) The XEDS maps for Cr-K and Te-L, respectively. (d) The high-resolution STEM-HAADF imaging for the cross-sectioned specimen of 1T-$CrTe_2$. (e) The ADF image acquired simultaneously with EELS mapping of the yellow rectangle area in Supplementary Figure 8 d. (f) The EELS elemental map for Cr-L signal using the energy loss range of 571-582 eV, mainly containing the Cr-L_3 edge (labeled in green in Supplementary Figure 7 f). (g) The EELS spectrum extracted from the whole region.

The EELS mapping in Supplementary Figure 8f can be considered to mainly contain the Cr-L signal, due to the Te-$M_{4,5}$ edge being a delayed one, even though it begins at 577 eV.

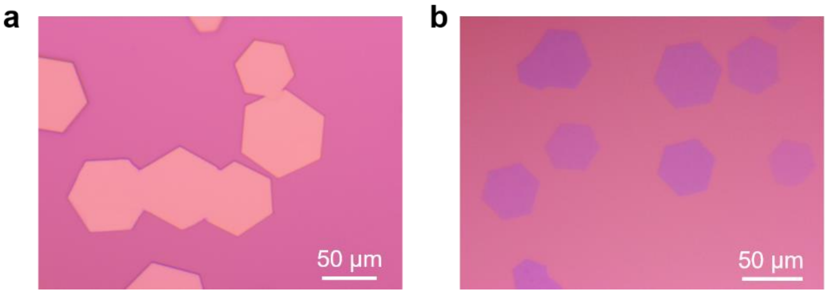

Supplementary Figure 9. Topographies and optical contrast images of 1T-$CrTe_2$ with the thickness of about 40.0 nm (a) and 3.0 nm (b), respectively.

It is worth noting that the VSM test can only obtain the averaged signal, and the thickness of the sample will significantly affect the signal obtained. Before processing the VSM/SQUID test, we statistically analyzed the thickness data of 1T-$CrTe_2$ samples on a large scale, and use the sample thickness with the dominate percentage to name

the sample.

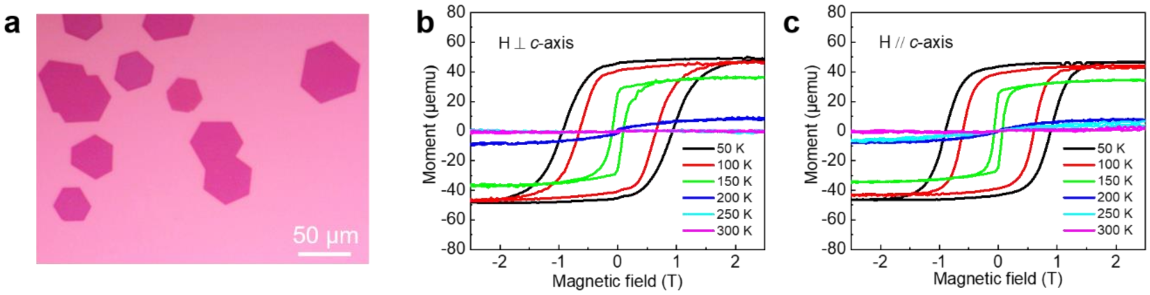

Supplementary Figure 10. SQUID measurement of 1T-$CrTe_2$ with thickness of about 10 nm. (a) Topographies and optical contrast image of 1T-$CrTe_2$ sample. (b, c) The corresponding temperature-dependent magnetic hysteresis loops under the magnetic field vertical (b) and parallel (c) to the *c*-axis of the samples, respectively.

For the sample with an intermediate thickness of 10.0 nm, the hysteresis loops resemble each other under parallel and vertical magnetic field, showing a magnetic-isotropy-like behavior, indicative of the critical thickness is approximately 10.0 nm

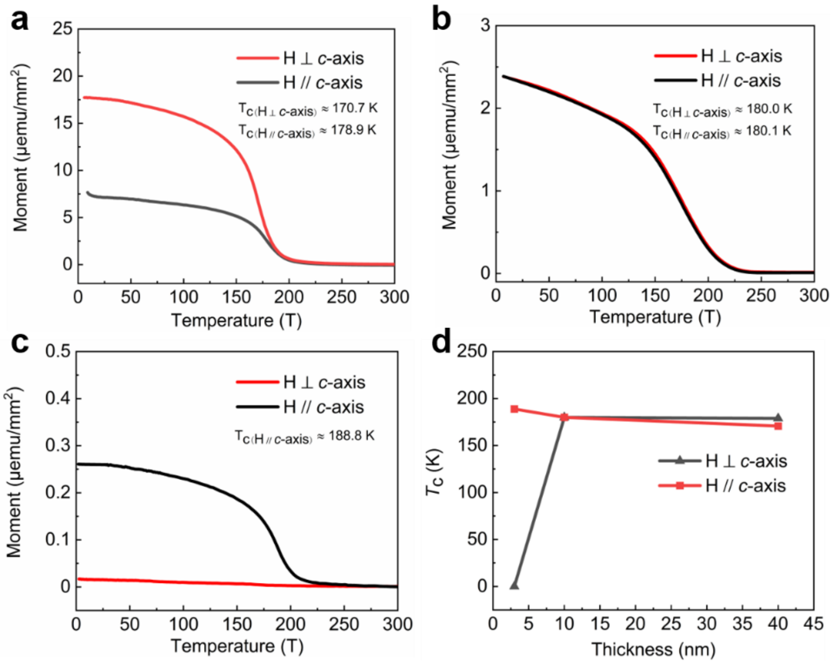

Supplementary Figure 11. M-T curve and corresponding T_c of 1T-$CrTe_2$ with three typical thicknesses. The tested samples have the thickness of about 40.0 nm (a), 10.0 nm (b), and 3.0 nm (c). Both the out-of-plane and in-plane magnetic fields were applied to the samples. (d) T_c as a function of thickness under out-of-plane and in-plane magnetic field, respectively. The cooling process was conducted under an external field of 1300 Oe.

For the magnetic field vertical to *c*-axis, results from three typical samples with varying thickness demonstrate a robust M-T relationship even down to the thickness of about

3.0 nm (Supplementary Figure 11a, b, c). The calculated T_c for 40.0 nm thick 1T-$CrTe_2$ crystals is ~ 170 K, while it is ~ 189 K in the thickness of 3.0 nm. The T_c increases as the sample thickness decreases (Supplementary Figure 11d)[7], this trend is different from the recent reports on other two-dimensional ferromagnetic materials[8,9]. For the in-plane magnetic field, the magnetic transition only occurs in the relatively thick samples (Supplementary Figure 11 a, b), with no surviving remanence when the thickness reduces to a few layers. As shown in Supplementary Figure 11d, the behavior of the sample in 3.0 nm forms a sharp contrast to those in 40.0 nm and 10.0 nm, which show a similar T_c of ~180K, consistent with the conclusions from the M-H curves. These results suggest novel magnetic properties in the CVD grown 1T-$CrTe_2$ crystals. Given its van der Waals nature and magneto-crystalline anisotropy, we expect the long-range FM order survives in the atomically thin film with the 2D limit breaking the Mermin–Wagner restriction[10].

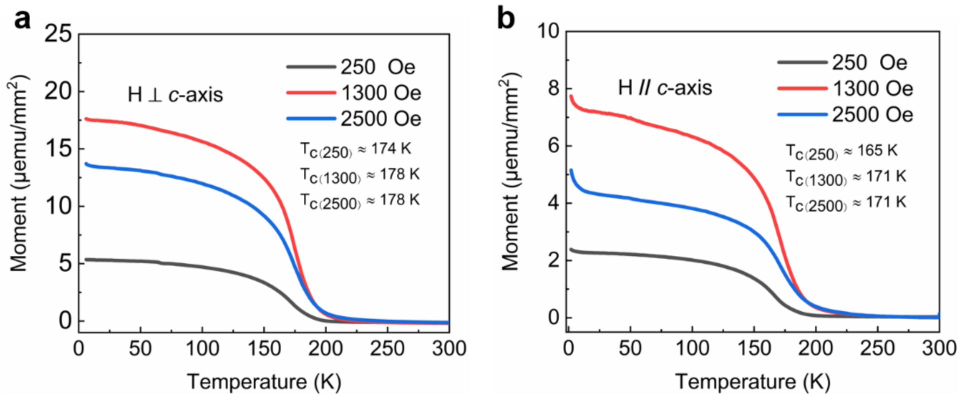

Supplementary Figure 12. M-T curve of 1T-$CrTe_2$ with thickness of ~ 40.0 nm under the magnetic field vertical (a) and parallel (b) to the c-axis of the crystal, respectively.

Noting that the magnetic field has significant effect on the criticality and a proper magnetic field is very important for determining the T_c. Thus, we compared the resulted M-T curve under different magnetic fields. Meanwhile, the magnetic moment from in-plane and out-of-plane has also been compared. Noting that the magnetic moment is normalized by the area. As shown in Supplementary Figure 12, the magnetic moment increases first and then decreases as the magnetic field increases at the same temperature. The extracted T_c at both in-plane and vertical magnetic field under a weaker magnetic field is slightly lower than that under a higher magnetic field. Thus, by comparing the values of magnetic moment and T_c under the three magnetic fields, we infer that 1300 Oe may be a suitable magnetic field.

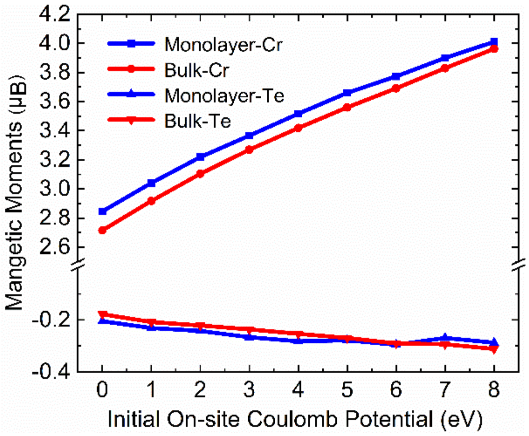

Supplementary Figure 13. DFT-calculated magnetic moments projected to each atom for monolayer and bulk 1T-$CrTe_2$ with the change of on-site Coulomb potentials. The *d* orbitals of Cr atoms couple with the *p* orbitals of Te atoms antiferromagnetically.

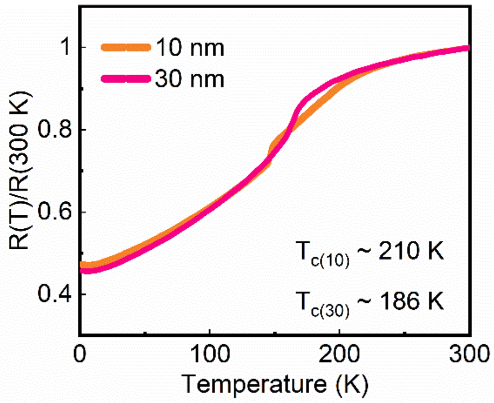

Supplementary Figure 14. Hall resistance as a function of temperature, measured on 10.0 nm and 30 nm thick device, respectively.

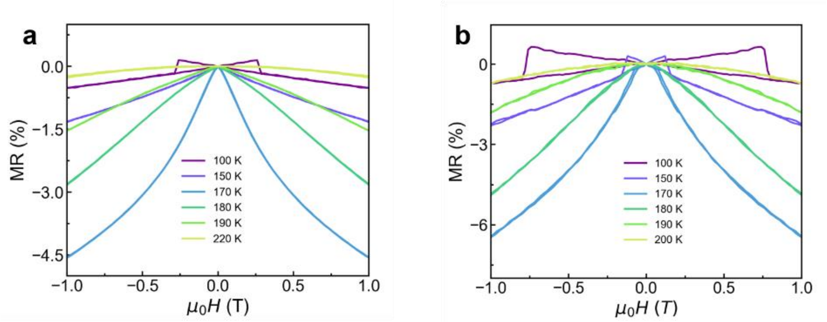

Supplementary Figure 15. Out-of-plane MR (a, b) measured on the 15.0 nm and 130.0 nm thick 1T-$CrTe_2$ Hall devices, respectively.

The magneto-resistance (MR) shows a butterfly-shaped hysteresis, with jumps at the spin-flip transition magnetic field, similar to the result of the 10.0 nm thick Hall device in the main text (Figure 4b).

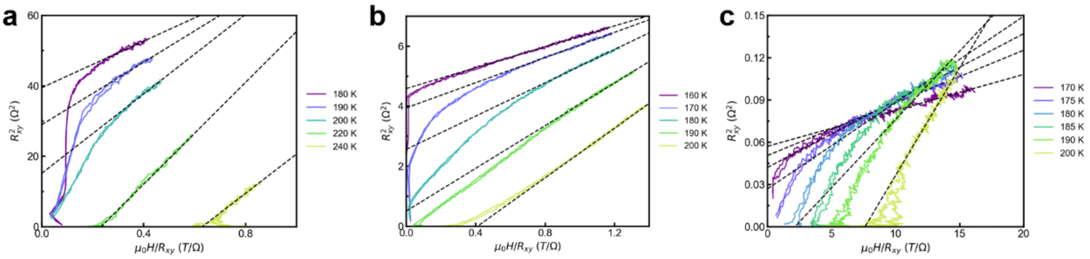

Supplementary Figure 16. Arrott plots[11] for a 10.0 nm, 15.0 nm, and 130.0 nm thick samples, respectively. (a) The obtained T_c is between 200 K and 220 K, thus we estimated T_c = 210 K. (b) The obtained T_c is between 190 K and 200 K, thus we estimated T_c = 193 K. (c) The obtained T_c is between 185 K and 190 K, thus we estimated T_c = 187 K.

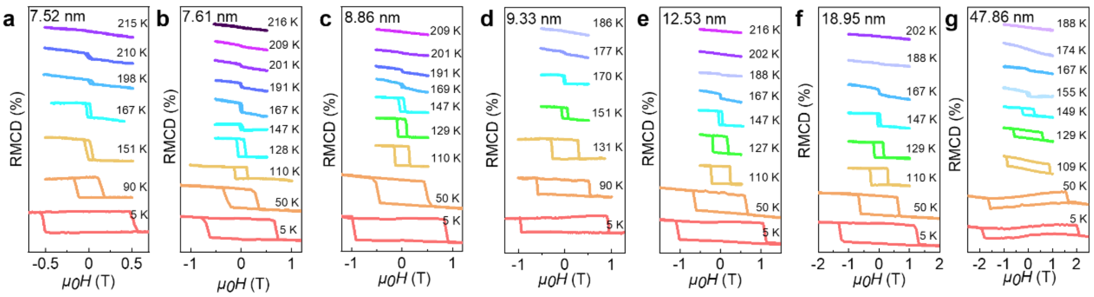

Supplementary Figure 17. RMCD measurements of 1T-$CrTe_2$ with different thicknesses under several fixed temperatures.

For all samples, the hysteresis loops shrink as the temperature going up and vanish at the magnetic transition point. The transition temperature from ferromagnetic phase to the paramagnetic state could be estimated from RMCD signal as a function of vertical magnetic field measured at several fixed temperatures.

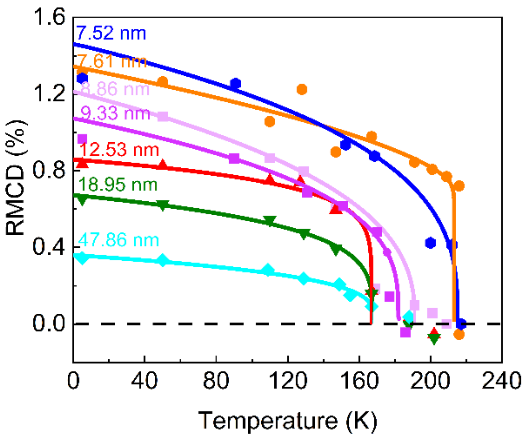

Supplementary Figure 18. Zero-field remanent RMCD signal as a function of temperature for samples with different thicknesses. The solid lines are the least square fitting results of primary data using the formula: $\alpha(1 - T/T_c)^{\beta}$. Zero RMCD signal is indicated by the dotted line.

In order to obtain the critical point stringently, the T_c was extracted from the plots of temperature-dependent remanence of RMCD signal at zero field (Supplementary Figure 18)[12]. Note the criticality fits are only accurate in the vicinity of the critical point, where the correlation length diverges. When we perform the fits, we start by fitting data very close to the Curie temperature and then progressively include more lower temperature data until the dates are all added. This cutoff ends up being $1 - T/T_c \lesssim 0.25$. The disappearance of magnetic signal in the 7.6 nm sample appears at around 212 K (Figure 4e in the main text), a prominent increase from the bulk value (d ~ 47.9 nm) of about 167 K (Figure 4f in the main text). This result, consistent with that of the previous SQUID and magneto-transport results, further confirms the anomalous thickness-dependent T_c in this material.

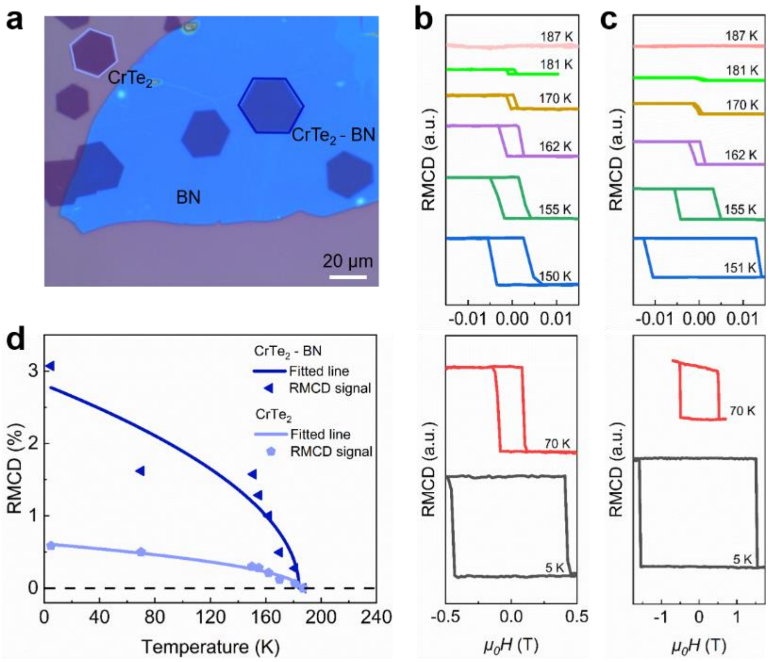

Supplementary Figure 19. The investigation of magnetic performance of 1T-$CrTe_2$ samples with and without encapsulation by RMCD. (a) OM image of the samples being tested. Samples with and without *h*-BN encapsulation are highlighted by a blue and light blue hexagon, respectively. (b, c) RMCD signal as a function of the out-of-plane magnetic field at different temperatures obtained in 1T-$CrTe_2$ domains without and with encapsulation. (d) Corresponding zero-field remanent RMCD signal as a function of temperature for samples being tested. The solid lines are the least square fitting results of primary data using the formula: $\alpha(1 - T/T_c)^{\beta}$. Zero RMCD signal is indicated by the black dotted line.

Supplementary References

1. Zhao, B. et al. Synthetic Control of Two-Dimensional $NiTe_2$ Single Crystals with Highly Uniform Thickness Distributions. *J. Am. Chem. Soc.* **140,** 14217-

14223 (2018).

2. Yang, P. et al. Thickness Tunable Wedding-Cake-like MoS_2 Flakes for High-Performance Optoelectronics. *ACS Nano* **13,** 3649-3658 (2019).
3. Najmaei, S, et al. Vapour phase growth and grain boundary structure of molybdenum disulphide atomic layers. *Nature Mater.* **12,** 754-759 (2013).
4. Freitas, D. C. et al. Ferromagnetism in layered metastable 1T-$CrTe_2$. *J. Phys. Condens. Matter* **27,** 176002 (2015).
5. Purbawati, A. et al. In-Plane Magnetic Domains and Néel-like Domain Walls in Thin Flakes of the Room Temperature $CrTe_2$ Van der Waals Ferromagnet. *ACS Appl. Mater. Interfaces* **12**, 30702-30710 (2020).
6. Li, J. et al. Synthesis of Ultrathin Metallic MTe_2 (M = V, Nb, Ta) Single-Crystalline Nanoplates. *Adv. Mater.* **30,** 1801043 (2018).
7. Wen, Y. et al. Tunable Room-Temperature Ferromagnetism in Two-Dimensional Cr_2Te_3. *Nano Lett.* **20**, 3130-3139 (2020).
8. Deng, Y. et al. Gate-tunable room-temperature ferromagnetism in two-dimensional Fe_3GeTe_2. *Nature* **563,** 94-99 (2018).
9. Gong, C. et al. Discovery of intrinsic ferromagnetism in two-dimensional van der Waals crystals. *Nature* **546,** 265-269 (2017).
10. Mermin, N. D. & Wagner, H. Absence of Ferromagnetism or Antiferromagnetism in One- or Two-Dimensional Isotropic Heisenberg Models. *Phys. Rev. Lett.* **17,** 1133-1136 (1966).
11. Arrott, A. Criterion for Ferromagnetism from Observations of Magnetic Isotherms. *Phys. Rev.* **108,** 1394-1396 (1957).
12. Stanley, H. E. Scaling, universality, and renormalization: Three pillars of modern critical phenomena. *Rev. Mod. Phys.* **71,** S358 (1999).
